# Internal nitrogen removal from sediments by the hybrid system of microbial fuel cells and submerged aquatic plants

**DOI:** 10.1371/journal.pone.0172757

**Published:** 2017-02-27

**Authors:** Peng Xu, En-Rong Xiao, Dan Xu, Yin Zhou, Feng He, Bi-Yun Liu, Lei Zeng, Zhen-Bin Wu

**Affiliations:** 1 State Key Laboratory of Freshwater Ecology and Biotechnology, Institute of Hydrobiology, Chinese Academy of Sciences, Wuhan, Hubei, China; 2 University of Chinese Academy of Sciences, Beijing, China; 3 College of Resources and Environmental Engineering, Wuhan University of Technology, Wuhan, Hubei, China; Natural Environment Research Council, UNITED KINGDOM

## Abstract

Sediment internal nitrogen release is a significant pollution source in the overlying water of aquatic ecosystems. This study aims to remove internal nitrogen in sediment-water microcosms by coupling sediment microbial fuel cells (SMFCs) with submerged aquatic plants. Twelve tanks including four treatments in triplicates were designed: open-circuit (SMFC-o), closed-circuit (SMFC-c), aquatic plants with open-circuit (P-SMFC-o) and aquatic plants with closed-circuit (P-SMFC-c). The changes in the bio-electrochemical characteristics of the nitrogen levels in overlying water, pore water, sediments, and aquatic plants were documented to explain the migration and transformation pathways of internal nitrogen. The results showed that both electrogenesis and aquatic plants could facilitate the mineralization of organic nitrogen in sediments. In SMFC, electrogenesis promoted the release of ammonium from the pore water, followed by the accumulation of ammonium and nitrate in the overlying water. The increased redox potential of sediments due to electrogenesis also contributed to higher levels of nitrate in overlying water when nitrification in pore water was facilitated and denitrification at the sediment-water interface was inhibited. When the aquatic plants were introduced into the closed-circuit SMFC, the internal ammonium assimilation by aquatic plants was advanced by electrogenesis; nitrification in pore water and denitrification in sediments were also promoted. These processes might result in the maximum decrease of internal nitrogen with low nitrogen levels in the overlying water despite the lower power production. The P-SMFC-c reduced 8.1%, 16.2%, 24.7%, and 25.3% of internal total nitrogen compared to SMFC-o on the 55th, 82th, 136th, and 190th days, respectively. The smaller number of *Nitrospira* and the larger number of *Bacillus* and *Pseudomonas* on the anodes via high throughput sequencing may account for strong mineralization and denitrification in the sediments under closed-circuit. The coupled P-SMFC system has shown good potential for the efficient removal of internal nitrogen.

## Introduction

Nitrogen (N) is one of the essential nutrients for plant growth but excess amounts of N create toxic algal blooms that cause eutrophication and deterioration of aquatic ecosystems by depleting oxygen in water bodies [1,2]. Besides the exogenous input, release from the sediments is generally regarded as the main source of N in the overlying water [3,4]. Therefore, in addition to controlling the exogenous input, measures for decreasing internal N loading (sum of all forms of N in sediments and pore water) should be taken to control the eutrophication process.

Sediment microbial fuel cells (SMFCs) have been used to harness bioelectricity from water-based ecosystems, in which electro-chemically active microorganisms metabolize biodegradable organic matter (OM) in sediments and generate electrons. The electrons are then transferred to the anode and flow to the cathode following the natural potential gradient. The protons and electrons react with oxygen at the cathode to form water [5,6]. Recently, SMFC has been used for bioremediation of the overlying water in the sediment-water system, specifically for N removal in wastewater [7,8], sediment remediation by biodegradation of organic pollutants [9], and phosphorus immobilization [10]. A previous study reported the cathode denitrification by SMFC in a cathode-chamber controlled anaerobic environment [11]. However, as the main source of N in overlying water, the influence of SMFC on the internal N is not well known. It is exigent to study the influence of SMFC on the migration and transformation of internal N to determine the potential of SMFC as a new technology for internal N removal.

In single-chamber microbial fuel cells (MFCs), the migration of NH_4_^+^ from the anode is always facilitated under electrogenesis [12]. In SMFC, the introduction of the anode as an electron acceptor can elevate the Eh value of the sediments [13]. The synthetic reaction at the cathode could also change dissolved oxygen (DO) and pH values of the overlying water, which is bound to influence the behavior of internal N in the sediments. The migration of internal NH_4_^+^ to the overlying water can also be facilitated in closed-circuit SMFCs, which may reduce internal N. At the same time, other measures should be considered to remove the accumulated N in the overlying water under electrogenesis.

Submerged aquatic plants as the main ecological groups of shallow lakes usually occupy the majority of the space between the water-phase and sediment-phase and regulate and control the cycles of material and energy [14]. Submerged aquatic plants play a vital role in stabilizing sediments, providing a large area for the attached growth of microorganisms, increasing sediment porosity, and providing a refuge to fish [1]. Furthermore, submerged aquatic plants can function well in internal NH_4_^+^ removal by assimilation [15] and stimulate the internal processes of nitrification and denitrification through changes in the microenvironment [16,17]. Many studies found that the combination of aquatic plants with MFCs improved power generation [18,19,20] and wastewater treatment efficiency [21,22], and increased the degradation rate of high-molecular-organic compounds in sediments [23]. However, the effects of the coupled P-SMFC system on internal N are not yet well-known, and the study of P-SMFC systems could provide a valuable reference for internal N removal by the combined phytoremediation and SMFC.

This study hypothesizes that electrogenesis could increase the N levels in the overlying water by decreasing the level of internal NH_4_^+^. Since aquatic plants function well in reducing internal N, they were introduced into SMFCs to investigate whether the internal N levels could be further reduced with alleviated N accumulation in the overlying water. We developed a comparative study to examine the influence of SMFCs and P-SMFCs on the migration and transformation of internal N and evaluate the effects on internal N in sediment-water microcosms.

## Materials and methods

### Ethics statement

This study was carried out in the indoor environment to simulate the sediment-water system at the Westlake workstation, located between the Qiantang River and the West Lake in Hangzhou city in east China’s Zhejiang Province (30°21'N 120°15'E). The aquatic plants (*V*. *spiralis*) used in this study are not considered endangered or threatened. The amount of sediments sampled from Westlake is relatively small. Therefore, the influence of the experiments on the natural environment is negligible. Necessary permits to conduct the study are granted by the owner of the workstation (the management office of Westlake waters).

### Configuration and setup of the experimental system

SMFCs used in the experiments were constructed using rectangular tanks made of glass with a total volume of 79.6 L (35×35×65 cm) and effective working volume of 73.5 L. Each tank was filled with 10 kg lake sediment to a depth of 15 cm and then filled with tap water to a depth of 45 cm. In SMFCs, non-catalyzed graphite felts (thickness of 0.5 cm) were used as electrodes. The felts were first soaked in 1 M HCl for 24 h, then washed with water before use. In every tank, three glass strips, 8.4-cm apart, were fixed at about 7 cm above the bottom of the tank. Three pieces of graphite felts with a total projected area of 1,575 cm^2^, each 35 cm long and 15 cm wide, were placed in the sediment vertically and fixed on the strips using plastic clips, then connected by a titanium wire (used as the anode). The cathode, which had the same total surface area (length 45 cm, width 35 cm) was placed vertically in the oxic water at a distance of 5 cm from the water-sediment interface and was fixed on the glass walls using clamps. The distance between the centers of the anode and cathode electrodes was 32.5 cm. Copper wires were used to connect the electrodes to the external circuit which was connected by a resistance of 100 Ω for SMFC-c and P-SMFC-c. In every tank, three Rhizon SMS samplers (Safe Biotech Co., Shanghai) for collecting pore water were placed horizontally in the sediments with a spacing of 5 cm, with the upper one being 3 cm below the sediment-water interface. The sediment samples were covered with black paper to prevent light from reaching them.

**Fig 1:**
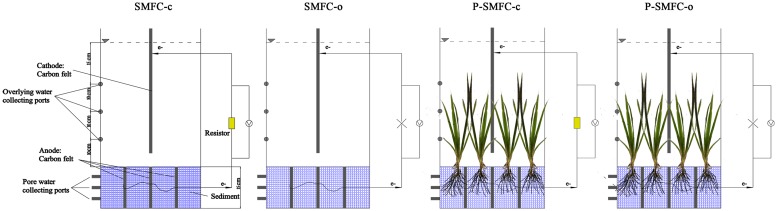
Schematic diagram of experimental devices including four treatments of SMFC-o, SMFC-c, P-SMFC-o, and P-SMFC-c.

The experimental system consisted of 12 tanks, including four treatments in triplicates: SMFC under open circuit (SMFC-o), SMFC under closed circuit (SMFC-c), aquatic plants with SMFC under open circuit (P-SMFC-o), and aquatic plants with SMFC under closed circuit (P-SMFC-c). Schematic diagram of the experimental device is shown in Fig 1. A total of four fluorescent lamps (30 W) were used to illuminate the tanks 12 h a day. The artificial illumination resulted in an average light intensity of 400 lux, which guaranteed the growth of the plants.

### Sediments, aquatic plants, and overlying water

Sediment samples were taken manually from the Xiaonan Lake of West Lake in Hangzhou city of China (30°23'N 120°14'E), using a Pedersen grab sampler. First, debris consisting of macrofauna and aquatic plants in sediments was manually removed using a 2-mm sieve, then the sediments were mixed using a ladle to obtain a homogeneous mixture. The initial properties of the sediments after the pre-treatment are listed in Table 1. As the dominant species in West Lake, submerged aquatic plants, *V*. *spiralis*, were transplanted into the P-SMFC-c and P-SMFC-o systems. The *V*. *spiralis* collected from the lake were washed to remove sediment residues; well-grown aquatic plants with similar root length (8–10 cm) and a similar number of green leaves (6–8) were selected; 12 strains with 15-cm leaf lengths were transplanted into the sediments in each tank. The experiment lasted for 194 d, sustaining the whole electricity production process and the growth of *V*. *spiralis*. To simulate the eutrophic lake rich in NO_3_^-^, KNO_3_ was added into the overlying water at a concentration of 2 mg/L on the 1th, 64th, and 145th day, respectively. The initial NH_4_^+^ concentration was 0.31 mg/L. Water lost due to evaporation and sampling was replenished with distilled water. In order to determine the influence of electrogenesis on the chemical forms and formation mechanisms of N at different time periods, the experiment was divided into three phases: phase I (days 1 to 55), phase II (days 64 to 136), and phase III (days 145 to 190) with static operation.

**Table 1:** Properties of pre-treated sediments used for experiments.

			Nitrogen concentrations (mg N/kg dry-weight)				
pH	OM (dt %)	Water content (wt %)	Total N	ON	NH_4_^+^	NO_3_^-^	NO_2_^-^
7.30±0.13	13.33±0.58	40.0±4.0	2215.2±29.7	2093.9±36.5	26.5±6.1	94.8±3.2	ND

The data was presented as mean value ± standard deviation. dt % and wt % represent the percentage of dry-weight and wet-weight, respectively.

### Sampling, analysis, and calculations

Open circuit voltage (OCV) and closed circuit voltage were recorded by paperless recorders (R6000, Jisame Electric Co., China) every 5 min. The cathode potentials were measured by positioning the Ag/AgCl reference electrodes near the cathode. The values from subtracting cell potential from cathode potentials were approximated as anode potentials. Anode potential was approximately equal to the sediment Eh value. Current density (mA/m^2^) and power density (mW/m^2^) were gauged based on the surface area of the anode. Ohm’s Law (U = IR, U: voltage, R: external resistance) was used to calculate the current (I). The resistances were changed from 90 kΩ to 100 Ω to conduct the polarization curve test during the stable period of voltage production, while stable voltages were automatically recorded. Power curves were obtained to describe the power density as a function of the current density.

The overlying water parameters such as dissolved oxygen (DO), temperature (T), and pH were measured using a Hach portable water quality analyzer (HQ30d, Hach Company, USA) at 5 cm beyond the interface. Mixed samplings of equal volume from three layers of overlying water were collected every 9 d to determine the ammonium (NH_4_^+^) and nitrate (NO_3_^-^) content. All pore water samples (10 mL) were collected every 27 d and analysed for TN, NH_4_^+^, and NO_3_^-^ contents. All samples were tested within 24 h according to the Chinese National Quality Standard and related national guidelines [24]. The air-drying was used to determine the water content (wt %) of pre-treated sediments. The OM content was determined by weighing the sample before and after combustion at 550°C for 4h. The pH of the sediments was measured using a pH meter (PHS-3C, China). K_2_S_2_O_8_ digestion method was used to measure TN in sediments [25]. An extracting solution of 1 mol/L KCl was used to extract NH_4_^+^ and NO_3_^-^ from sediments [26], followed by standard laboratory analysis. All forms of internal N are considered the sum of the forms in sediments and pore water. Internal organic nitrogen (ON) was approximated by subtracting the internal NH_4_^+^ and NO_3_^-^ from internal TN using the following equation:

$$TN=NH_{4}{}^{+}+NO_{3}{}^{-}+ON$$ (1)

According to the initial properties of the sediments presented in Table 1, the water content (wt %) of sediments was 40.0%. Therefore, each tank contained 10 kg sediments with 4 L water and 6 kg dry sediments. Eq (2) was used to calculate internal TN:

$$I_{TN}=V_{W}P_{TN}+M_{S}S_{TN}$$ (2)

where I_TN_ is internal TN, V_W_ is the internal water volume (4 L in this study), P_TN_ is the TN concentration in pore water, M_S_ is the dry sediment weight (6 kg in this study), and S_TN_ is the TN contents in sediments. The growth of *V*. *spiralis* was evaluated by measuring the maximum stem and leaf lengths. *V*. *spiralis* was harvested and rinsed with water to obtain its fresh weight. 12 Treated aquatic plants were dried at 80°C for 48 h and then weighed to measure the dry weight. The nitrogen content of dried aquatic plants was measured using the H_2_SO_4_-H_2_O_2_-Colorimetric method [27].

At the end of the experiment, the extraction of genomic DNA from the anode samples from 12 tanks was conducted. Prior to extraction, the sediments on the anode surface were washed out using sterile water, and anode-attached biofilms were acquired by scraping the anode electrodes using a sterile razor blade. An E.Z.N.A.^®^ DNA Isolation Kit (Omega Biotek, USA) was used to extract the genomic DNA. Illumina high-throughput sequencing using highly pure genomic DNA (A260/A280 ≈ 1.8) was conducted by Hengchuang Biotechnology Company (Shenzhen, China). Biodiversity and richness indices were obtained using the QIIME software package. Clustering similar sequences into operational taxonomic units (OTUs) was based on 3% dissimilarity. Ribosomal Database Project (RDP) was chosen for taxonomic classification, with a bootstrap cut-off of 80%, down to the phylum, class, order, family, and genus level. Cluster and Treeview programs were used to construct the taxon heatmap.

### Statistical analysis

All data are presented as the mean value and standard deviation (SD). The SPSS software was used to evaluate the significance of differences by a two-way ANOVA and Fisher’s LSD tests at the 0.05 probability level. Correlation analysis was adopted to evaluate the relationships between nitrogen and influential factors such as pH, DO, T, and Eh, with *p* < 0.05 representing statistically significant correlations.

## Results and discussions

### Electrochemical characteristics of SMFCs and P-SMFCs

**Fig 2:**
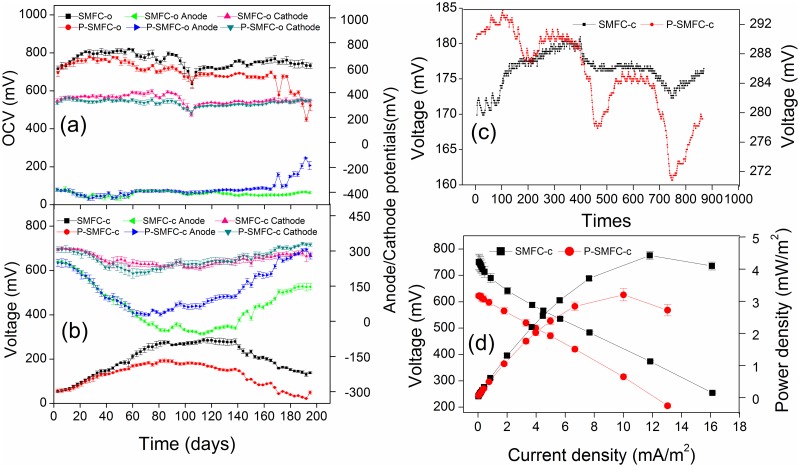
Bio-electrochemical characteristics of SMFCs and P-SMFCs. (a) Open circuit voltage and cathode/anode potentials. (b) Closed circuit voltage and cathode/anode potentials. (c) Closed circuit voltage variation in 72 h, 864 time measurements, recorded at 5-minute intervals from 0:00 am on the 100th d. (d) Polarization and power density curves. In (a) and (b), each point stands for the mean value recorded in previous three days.

Voltage variations were observed in a parabolic curve under external resistances of 100Ω. As shown in Fig 2b, electrogenesis could be divided into three phases with the start-up in phase I, stable production during phase II, and electricity attenuation in phase III. Voltages of 54.9 mV and 56.9 mV were documented on the first day of the operation of SMFC-c and P-SMFC-c, respectively. The voltage maintained the sluggish growth in phase I, attributed to the slow formation of electrochemically active biofilm on the anode surfaces. The difference in the voltage/anodic potential between SMFC-c and P-SMFC-c was not significant. During the stable phase, relatively lower voltage generation was observed for P-SMFC-c than SMFC-c when maximum voltages of 287 mV and 193 mV were documented, respectively. With the depletion of biodegradable carbon sources that maintained the normal physiological activity of microorganisms, significant decreases in voltage were detected afterward. The difference in the voltage between SMFC-c and P-SMFC-c was still significant. During the whole operation, the mean voltage was decreased by 75 mV in the presence of aquatic plants. The visible decrement in power density was noticed with the addition of aquatic plants when maximum power densities were observed for SMFC-c (4.42 mW/m^2^ at 200 Ω) and P-SMFC-c (3.16 mW/m^2^ at 200 Ω) (Fig 2d). The plant microbial fuel cell (PMFC) is generally defined as a new technology that can potentially provide renewable and sustainable energy from rhizodeposition utilized as a carbon source by electrochemically active bacteria (EAB) [19,20]. Previous studies showed that the rhizodeposition could be used as the only electron donor for power generation in MFCs [19,20]. A previous study demonstrated that the electrical power output of an SMFC was found to be a factor of 7 higher in the presence of actively growing plants [18]. However, in this study, the submerged aquatic plant *V*. *spiralis* did not improve the power generation of SMFC because the low amounts of exudates compared with the emergent aquatic plants with large amounts of root exudates used in previous studies were not enough to affect the power generation when the sediments were the main sources responsible for the electrogenic activity.

The decrement in the electrogenic capacity in the presence of aquatic plants can be validated by lower OCV as shown in Fig 2a, implying a decreased biopotential maintenance and power production. Lower power generation may be attributed to higher internal resistance measured from the slope of the polarization curve as seen in Fig 2d with 167 Ω and 193 Ω for SMFC-c and P-SMFC-c, respectively. Higher anodic resistance and pH gradient resistance may occur in the presence of aquatic plants. A previous study analyzed the quantitative imaging of radial oxygen loss (ROL) from *V*. *spiralis* roots with a fluorescent planar optode and found that oxygen release to the rhizosphere of *V*. *spiralis* was much higher than many other macrophyte species [28]. The local current generated by the competition for electrons by oxygen released from the roots of aquatic plants in the sediment layer results in a lower electron transfer rate and higher anodic charge transfer resistance [29,30]. Oxygen loss from photosynthesis could be further proved by the circadian oscillation phenomenon in P-SMFC-c during the 72-h period when the lower voltage was documented during daytime (Fig 2c). Acidification of sediments resulted in the accumulation of protons (S1 Table) and higher pH gradient resistance was formed when alkalization of overlying water occurred [31]. The introduction of SMFC-c accompanied by electron flow from sediments to water column increased the sediment Eh by 474 mV when mean anode potential values of 63 mV and -411 mV were documented in SMFC-c and SMFC-o, respectively, this result was in agreement with a previous study [13]. The highest sediment Eh was achieved by the coupled system when 550 mV was increased compared to SMFC-o.

### Bacterial communities on anodes

**Fig 3:**
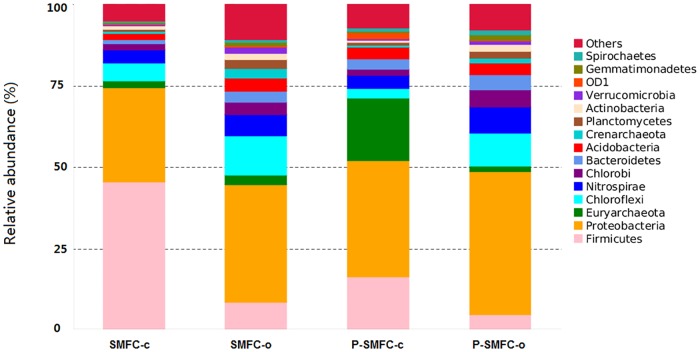
Taxonomic classification of bacterial sequences from communities on anode films at the phylum level.

*Proteobacteria*, the most abundant species in the system, accounted for 28.8%, 36.1%, 35.7%, and 43.8% of the total relative abundance in SMFC-c, SMFC-o, P-SMFC-c, and P-SMFC-o, respectively (Fig 3). The SMFC under closed-circuit allows for the capture of electrons by the anode, resulting in a shift in the bacterial community, a phenomenon that has also been observed in conventional SMFCs [32]. Within *Proteobacteria* phylum, *δ-Proteobacteria* obtained dominant enrichment (15.2% and 24.8% of the total class sequences versus 13.1% and 16.1% of that under open-circuit, S2 Table) on the anodes of SMFC-c and P-SMFC-c. *Firmicutes* constituted relatively higher shares of 45.7% and 16.5% in SMFC-c and P-SMFC-c, respectively, compared with those in open-circuit (8.7% and 4.9% in SMFC-o and P-SMFC-o, respectively). *Firmicutes*, involved in power generation in the glucose-fed microbial fuel cells (MFCs), were also found in another study [33]. However, the reason for the high abundance of *Firmicutes* in SMFC-c without glucose addition in this study remains unclear.

**Fig 4:**
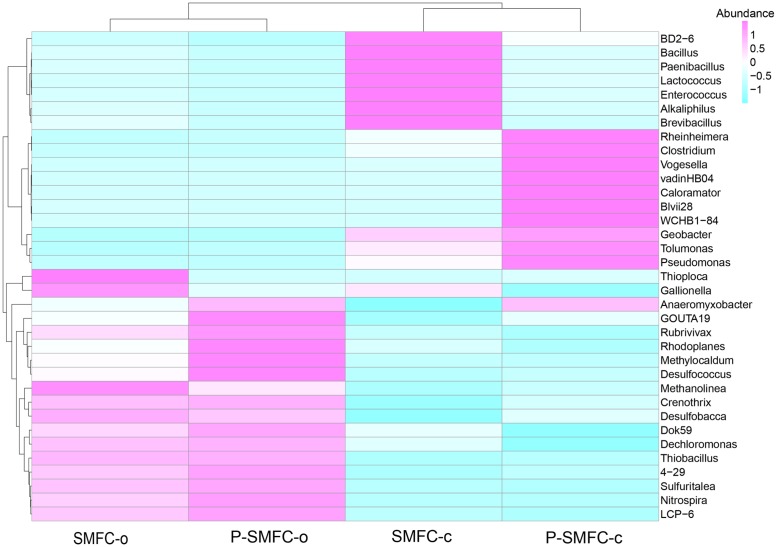
Heatmap of the samples tested for classified bacterial genera with the relative abundance of top 35 based on illumina sequencing.

Relative percentages of the top 35 genera are listed in Fig 4 at the genus level. *Bacillus* and *Enterococcus*, belonging to *Firmicutes*, were enriched significantly on the anode of SMFC-c, but showed relatively lower concentrations on the anode of P-SMFC-c. The two genera were proven to be related to electricity production with high electrochemical activity [34,35]. The electrogenic bacteria of *Clostridium* (belonging to *Firmicutes*) [36], *Geobacter* (belonging to *δ-Proteobacteria*) [37], and *Pseudomonas* (belonging to *δ-Proteobacteria*) [38] were significantly enriched on the anodes of P-SMFC-c. Among these electrogenic genera, *Bacillus* and *Geobacter* were the dominant ones. The relative percentages of *Bacillus* (*Geobacter*) were 22.5% (7.4%) and 4.4% (10.3%) on the anodes of SMFC-c and P-SMFC-c, respectively.

### Migration and transformation of internal N in sediment-water microcosms

**Fig 5:**
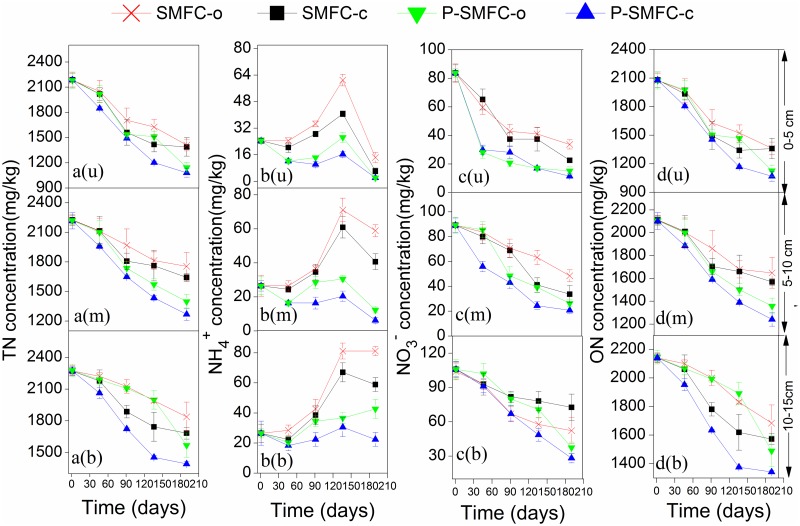
Nitrogen concentration changes in sediments. (a) TN. (b) NH4+. (c) NO3-. u, m, and b represent the upper, middle, and bottom layer, respectively.

**Fig 6:**
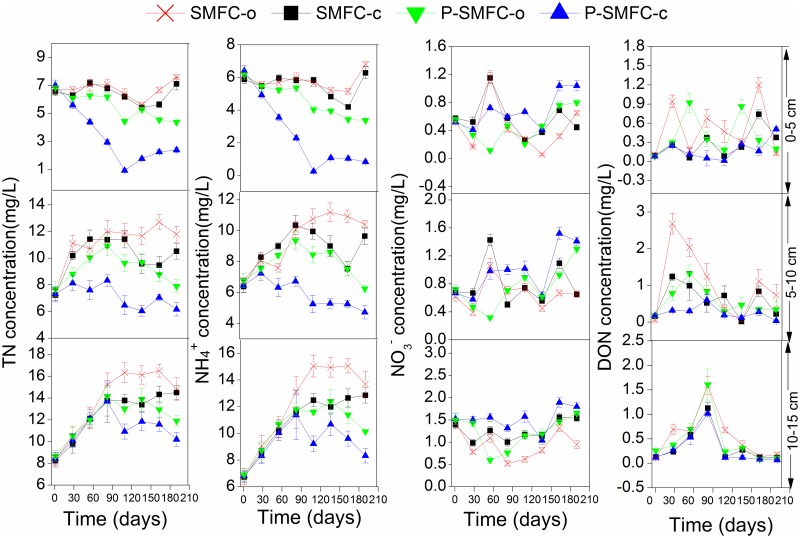
Nitrogen changes in pore water during 190 d. (a) TN. (b) NH_4_^+^. (c) NO_3_^-^. u, m, and b represent upper, middle, and bottom layer, respectively.

**Fig 7:**
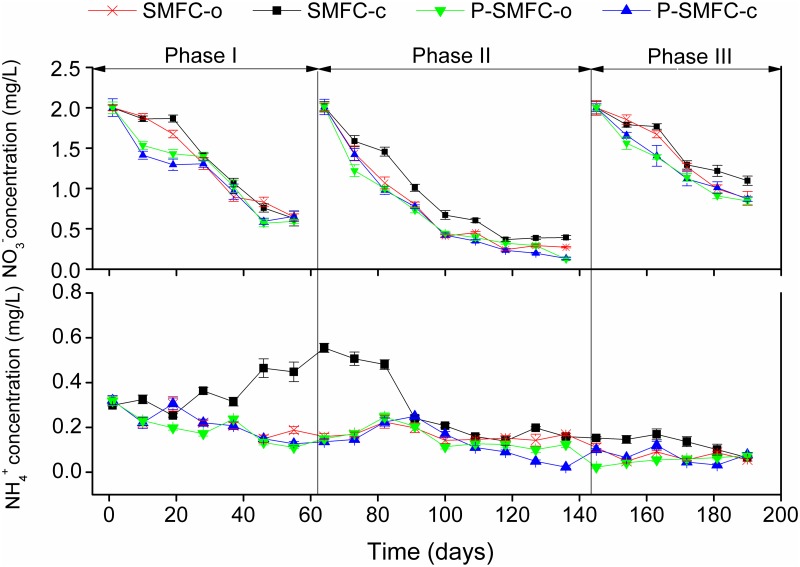
NO3- (a) and NH4+ (b) changes in overlying water during three phases of operation.

Consistent with other studies, the TN removal in sediments (Fig 5a) was through a series of microbial cycles: the constant mineralization of ON dominated in TN (Fig 5d), migration of NH_4_^+^ generated from the mineralization to the water column as insignificant NH_4_^+^ accumulation was observed (Fig 5b), and denitrification (Fig 5c) in sediments [39,40]. NH_4_^+^ in pore water was the main component of TN (Fig 6). Internal NH_4_^+^ was constantly generated in SMFC-c and SMFC-o in early stages of the experiment, followed by a significant decrease due to weakened mineralization. Initial NH_4_^+^ in overlying water was mainly from pore water along the concentration gradient (Fig 7b). The constant decrease of NH_4_^+^ in overlying water was attributed to the decreasing release and increasing nitrification over time with increasing DO levels (a in S1 Fig). Denitrification at the sediment-water interface resulted in a constant decrease of NO_3_^-^ in the overlying water (Fig 7a) [41]. The process did not influence NO_3_^-^ levels in sediments and pore water significantly.

### Influence of SMFC on the migration and transformation of internal N

**Fig 8:**
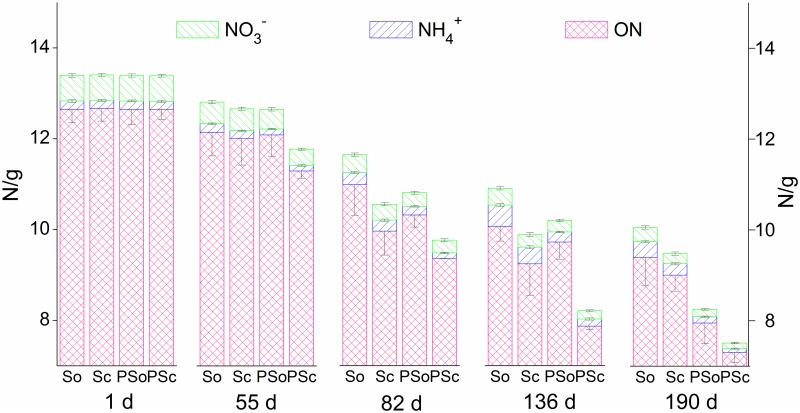
Different forms of internal nitrogen including ON, NH4+, and NO3- changes over time. So, Sc, PSo, and PSc represent SMFC-o, SMFC-c, P-SMFC-o, and P-SMFC-c, respectively.

During the start-up period of electrogenesis, the low power generation did not influence the internal NH_4_^+^ levels when SMFC-c and SMFC-o were compared. A previous study showed that a lower DO in overlying water could facilitate the NH_4_^+^ release from sediments and inhibit nitrification [42]. Therefore, lower DO levels in the overlying water of SMFC-c due to the consumed synthetic reaction (Table 2) might result in significant NH_4_^+^ accumulation (*p* < 0.05) (Fig 7b). During the periods of II and III with stable power generation followed by the drop, the stronger mineralization of ON and internal NH_4_^+^ removal were tightly linked in SMFC-c (Fig 8). The facilitated migration from the sediments to the overlying water was the main channel to be removed. A previous study reported that in the single-chamber MFC, N losses from the anode increased as a result of elevated pH near the cathode, which drove the ammonium volatilization with the conversion of ammonium ion to the more volatile ammonium species [12]. In this study, the accumulated quantity of NH_4_^+^ in the overlying water was significantly lower than the internal decrement of NH_4_^+^. Therefore, the same mechanism also predominantly drove the anodic NH_4_^+^ removal in SMFC. The NO_3_^-^ generation in pore water was another access to be linked with internal NH_4_^+^ reduction (Fig 6c). A previous study showed that NH_4_^+^ in the anode chamber could be used to generate electricity through aerobic ammonium oxidation coupled with anammox in a similar rotating-cathode single MFC; the NO_2_^-^ and NO_3_^-^ as the intermediates were the main products, accounting for 69.3% and 14.4% of NH_4_^+^ removal [43]. However, in this study, NO_2_^-^ was not detected in pore water and sediments and the internal NH_4_^+^ was not involved in electricity generation. The NO_3_^-^ generation in pore water was caused by strong nitrification with higher sediment Eh being conducive to the growth of aerobic bacteria [44]. A previous study of cathodic denitrification in SMFC was conducted in a strictly anaerobic environment where electrons were used by autotrophic denitrifying bacteria (ADB) formed on the surface of the cathodic electrode [11]. However, in this study, NO_3_^-^ accumulated in the overlying water of SMFC-c during phases II and III. The cathodic denitrification was difficult to determine when the oxygen-enriched environment was not conducive to the growth of ADB when the air-cathode was adopted, even at the sediment-water interface, the DO level was higher than 2 mg/L (Table 2). Stronger nitrification during these two phases also contributed to the transformation of NH_4_^+^ to NO_3_^-^. Furthermore, the increased thickness of the oxygenated sediment layer could inhibit denitrification at the sediment-water interface [45]. Therefore, the influence of internal N on NO_3_^-^ and NH_4_^+^ should not be neglected based on the increased sediment Eh and internal NH_4_^+^ migration. During the whole operation, NH_4_^+^ and ON in sediments were influenced by electrogenesis more than those in pore water, when 47.0 (3.09) mg of mean internal NH_4_^+^ and 470.2 (1.16) mg of mean internal ON were reduced in sediments (pore water) (Fig 8). These results suggest that electrogenesis promotes sediment mineralization and that the migration of NH_4_^+^ to the liquid pore water is facilitated and followed by removal through volatilization and nitrification.

**Table 2:** Physicochemical characteristics of overlying water during three phases of operation.

	Phase I (1–55 d)				Phase II (64–136 d)				Phase III (145–190 d)			
Parameter	SMFC-o	SMFC-c	P-SMFC-o	P-SMFC-c	SMFC-o	SMFC-c	P-SMFC-o	P-SMFC-c	SMFC-o	SMFC-c	P-SMFC-o	P-SMFC-c
pH	7.93±0.22b	7.82±0.31c	8.14±0.32a	8.16±0.37a	8.24±0.27c	8.45±0.28b	8.61±0.19a	8.66±0.20a	8.56±0.18b	8.55±0.19b	8.70±0.22a	8.72±0.17a
DO(mg/L)	2.41±0.78a	2.04±0.54b	2.41±0.84a	2.41±0.94a	2.99±0.75b	2.83±0.40b	3.44±0.70a	3.40±0.56a	3.91±0.32a	3.53±0.31b	4.06±0.59a	4.05±0.21a
T(°C)	23.28±4.44	23.36±4.53	23.30±4.43	23.23±4.38	26.64±0.78	26.60±1.01	26.70±0.77	26.57±0.85	21.26±1.82	21.84±0.96	21.71±1.97	21.38±1.51
NO_3_^-^(mg/L)	1.32±0.55a	1.37±0.56a	1.22±0.52b	1.17±0.49b	0.77±0.62b	0.93±0.61a	0.72±0.60bc	0.71±0.64c	1.44±0.46b	1.53±0.37a	1.31±0.44c	1.34±0.43c
NH_4_^+^(mg/L)	0.23±0.06b	0.35±0.08a	0.20±0.07c	0.22±0.07bc	0.17±0.03b	0.29±0.17a	0.15±0.05c	0.13±0.07d	0.075±0.025b	0.128±0.038a	0.053±0.018c	0.074±0.033b

n = 6, 8, 5 for parameters in phase I, II, and III, respectively. The data was presented as mean value ± standard deviation. Different lowercase is defined as significantly different.

Significant removal of NO_3_^-^ (*p* < 0.05) in sediments was observed in SMFC-c compared to SMFC-o with 5.7%, 12.0%, and 13.3% in the upper, middle, and bottom layers, respectively. A Previous study showed that denitrification and substrate removal in MFC could simultaneously occur at the anode where the power was generated [46]. In other words, denitrification could be stimulated under electrogenesis. Denitrification demanding OM as a carbon source could be achieved when the circuit was closed. Electrons released by the oxidation of OM and transferred to the anode were taken from the anode by another organism for NO_3_^-^ reduction [47]. The relative abundance of *Nitrospirae* on the anode of SMFC-c was 4.0%, which was significantly lower than that of SMFC-o (6.6%). *Nitrospira*, affiliated to *Nitrospirae*, was significantly more abundant in SMFC-o, with a relative abundance of 0.7%. *Nitrospira* is known for supporting chemolithotrophic growth by the oxidation of nitrite to nitrate [48], implying an increase in the denitrification process in SMFC-c. The EAB *Bacillus*, related to denitrifying bacteria [49], may also explain the improvement of denitrification in sediments. Besides, the enrichment of denitrifying bacteria on the anodes may also accelerate nitrification in pore water by the consumption of NO_3_^-^.

Removal rates of internal TN by SMFC-c compared to SMFC-o were 1.2%, 9.4%, 9.3%, and 5.8% on the 55th, 82th, 136th, and 190th days, respectively (Fig 8). The higher internal TN removal rate was during the period of high power generation, the more significant mineralization, volatilization of NH_4_^+^, and nitrification-denitrification by SMFC-c were. Furthermore, the SMFC-c had a greater influence on the internal N in the middle and bottom layers (Tables 3 and 4), Oxygen as an electron acceptor could also facilitate the mineralization and nitrification-denitrification processes. At the sediment-water interface, high oxygen levels might attenuate the role of SMFC on the internal N removal.

**Table 3:** Characteristics of TN, NH4+, and NO3- in pore water.

	Upper layer (5–10 cm)				Middle layer (5–10 cm)				Bottom layer (10–15 cm)			
Parameter	SMFC-o	SMFC-c	P-SMFC-o	P-SMFC-c	SMFC-o	SMFC-c	P-SMFC-o	P-SMFC-c	SMFC-o	SMFC-c	P-SMFC-o	P-SMFC-c
TN(mg/L)	6.72±0.58a	6.39±0.64b	5.50±0.97c	3.40±2.08d	11.16±1.61a	10.15±1.42b	9.19±1.10c	7.14±0.87d	13.77±3.06a	12.49±2.30b	12.14±1.83b	11.12±1.58c
Re (%)		4.9	18.2	49.4		9.0	17.7	36.0		9.3	11.9	19.3
NH_4_^+^(mg/L)	5.77±0.53a	5.54±0.69b	4.63±1.06c	2.47±2.19d	9.47±1.72a	8.77±1.32b	7.87±1.03c	5.91±0.89d	12.24±3.13a	10.89±2.22b	10.46±1.83b	9.22±1.46c
Re (%)		4.0	19.8	57.2		7.4	16.9	37.6		11.0	14.5	24.6
NO_3_^-^(mg/L)	0.45±0.35c	0.58±0.27b	0.46±0.24c	0.68±0.25a	0.66±0.20c	0.79±0.31b	0.74±0.30b	0.98±0.35a	0.94±0.33c	1.26±0.22b	1.22±0.37b	1.53±0.26a
ON(mg/L)	0.50±0.41a	0.28±0.23c	0.40±0.31b	0.18±0.16d	1.03±0.95a	0.59±0.43b	0.57±0.39b	0.25±0.17c	0.55±0.49a	0.34±0.35b	0.46±0.50a	0.29±0.33b
Re (%)		44.8	19.7	64.3		43.0	44.4	75.5		38.7	16.7	47.8
NO_2_^-^(mg/L)	ND	ND	ND	ND	ND	ND	ND	ND	ND	ND	ND	ND

n = 8 for parameters. The data was presented as mean value ± standard deviation. Re stands for the removal efficiency compared with SMFC-o. Different lowercase is defined as significantly different. ND, not detectable. Detection limit = 0.0001 mg/L.

**Table 4:** Characteristics of TN, NH4+, and NO3- in sediments.

	Upper layer (5–10 cm)				Middle layer (5–10 cm)				Bottom layer (10–15 cm)			
Parameter	SMFC-o	SMFC-c	P-SMFC-o	P-SMFC-c	SMFC-o	SMFC-c	P-SMFC-o	P-SMFC-c	SMFC-o	SMFC-c	P-SMFC-o	P-SMFC-c
TN(mg/kg)	1795.6±316.1a	1715.4±365.6a	1678.9±418.8ab	1562.2±457.5b	1975.3±197.2a	1911.1±247.9ab	1805.7±348.7bc	1705.8±385.0c	2089.6±177.0a	1952.5±260.9b	2028.1±276.2ab	1781.6±381.4c
Re (%)		4.5	6.5	13.0		3.2	8.6	13.6		6.6	2.9	14.7
NH_4_^+^(mg/kg)	31.8±17.8a	24.1±12.5b	15.9±9.9c	13.1±8.2d	43.9±20.2a	37.4±14.7b	22.8±8.0c	17.2±7.4d	52.0±27.4a	42.7±19.7b	32.2±8.8c	24.1±4.6d
Re (%)		24.3	49.8	58.7		14.8	48.0	60.9		17.9	38.2	53.8
NO_3_^-^(mg/kg)	52.2±20.0a	49.3±24.6a	33.0±28.8b	34.1±28.8b	71.1±16.3a	62.6±24.2b	57.8±28.2b	46.7±27.7c	86.3±13.3a	74.8±22.6bc	79.2±27.6ab	68.1±31.4c
Re (%)		5.7	36.8	34.7		12.0	18.7	34.3		13.3	8.1	21.0
ON(mg/kg)	1711.7±300.3a	1642.1±342.3a	1630.0±391.5ab	1515.4±425.9b	1860.3±199.9a	1811.1±236.0ab	1725.0±319.5bc	1642.0±352.6c	1951.3±191.1a	1835.0±256.6b	1916.7±256.6ab	1689.4±351.9c
Re (%)		4.1	4.8	11.5		2.6	7.3	11.7		6.0	1.8	13.4
NO_2_^-^(mg/L)	ND	ND	ND	ND	ND	ND	ND	ND	ND	ND	ND	ND

n = 5 for parameters. The data was presented as mean value ±standard deviation. Re stands for the removal efficiency compared with SMFC-o. Different lowercase is defined as significantly different. ND, not detectable. Detection limit = 0.004 mg/kg.

### Influence of aquatic plants on the migration and transformation of internal N

The reductions of NH_4_^+^ and ON by *V*. *spiralis* in the three layers of pore water and sediments were also tightly coupled (Tables 3 and 4). The processes of ammonification, plant assimilation, and nitrification may be stimulated in pore water by *V*. *spiralis* (Fig 6). As the main bioavailable component, the root assimilation may contribute to NH_4_^+^ reduction when the concentration of bioavailable NH_4_^+^ in sediment pore water is much higher than that in overlying water [15]. The *V*. *spiralis* performed certain effect of NO_3_^-^ reduction just during the early colonization in sediments in the upper and middle layers of pore water (Fig 6c). The reasons might be as followes: uptake of NO_3_^-^ is more difficult than NH_4_^+^ because of the relatively higher energy consumption during the assimilation process [50]; besides, nitrification as a central process linking mineralization and potential N loss via denitrification could be facilitated in pore water when a suitable environment for stronger nitrification was formed in the presence of plant ROL [17]. In this study, the facilitated nitrification was also significantly observed after the initial colonization period. Previous studies found that the competition between the root assimilation of NH_4_^+^ and microbial processes around the rhizosphere often results in decreased nitrification [51,52]. However, the plant uptake was not at the level of limiting nitrifiers and the competition for NH_4_^+^ in the pore water between bacteria and the roots was attenuated in this study. Besides, NH_4_^+^ generated from ON mineralization would serve as the electron donor to support the growth of nitrifiers through the ammonia oxidation process [39].

A previous study examined the short-term changes in pore water chemistry during the early colonization by *V*. *spiralis* and found that NO_3_^-^ levels initially accumulated in pore water and then decreased due to enhanced denitrification [51]. However, in this study, the aquatic plants enhanced the NO_3_^-^ removal in sediments by 36.8% (18.7%, 8.1%) in the upper (middle, bottom) layer. The facilitated NO_3_^-^ removal by *V*. *spiralis* in P-SMFC-o was in the solid media of sediments, not in the pore water because denitrification occurs in sediments rather than in pore water owing to a denser colonization with denitrifiers and higher amounts of OM. The root excretion of exudates served as a carbon source to promote denitrification, when *Rhodoplanes* (belonging to *Rhodobacter*), a short-chain fatty acid-utilizing facultative denitrifier, was significantly enriched at the anode of P-SMFC-o [53].

P-SMFC-o played a more significant role on the internal N in the upper and middle layers (Tables 3 and 4), because of the length of the roots observed in the experiment. The measured oxygen penetration depths by *V*. *spiralis* from root tips were several millimeters with an extension of the incubation period [28]. A higher internal TN reduction rate was achieved by P-SMFC-o than by SMFC-c, however, significantly enhanced internal N removal by P-SMFC-o was just achieved in the later period of the experiment.

### Influence of P-SMFC on the migration and transformation of internal N

The accumulation of NH_4_^+^ in the overlying water due to electrogenesis in SMFC-c was significantly alleviated in the presence of aquatic plants when the mean concentrations of NH_4_^+^ (NO_3_^-^) were 0.22(1.17), 0.13(0.71), and 0.074(1.34) mg/L for P-SMFC-c in phases I, II, and III, respectively. Plant assimilation and microbial transformation might directly lead to smaller amounts of NO_3_^-^ and NH_4_^+^ in overlying water [15]. This study indicated that a combination of *V*. *spiralis* and SMFC further increased the internal N removal compared with SMFC-c and P-SMFC-o. The Eh value of the sediments in P-SMFC-c was significantly higher than that in SMFC-c, suggesting that the interactions between plant rhizosphere and SMFC anode were synergistic because of the strongest mineralization and nitrification-denitrification processes.

In P-SMFC-c, strong mineralization of internal ON was followed by a reduction of internal NH_4_^+^ when the removal efficiencies were 57.2% (37.6%, 24.6%) and 58.7% (60.9%, 53.8%) in the upper (middle, bottom) layer of the pore water and sediments. Although electrogenesis did not have a significant effect on the morphological characteristics of *V*. *spiralis* at the end of the experiment (S3 Table), the N content of *V*. *spiralis* in P-SMFC-c (291.0 mg) was significantly higher than that in P-SMFC-o (243.6 mg) (*p* < 0.05). This result implies that a greater amount of internal NH_4_^+^ is enriched in *V*. *spiralis* via assimilation when electrogenesis facilitates the release of NH_4_^+^ fixed on the minerals of the sediments to the pore water.

The highest NO_3_^-^ level in the pore water was consistent with the sediment Eh in P-SMFC-c, implying that coexistence of oxygen and the anode as electron acceptors could increase nitrification in the pore water. Previous studies showed that the aerobic oxidation of OM could minimize the thickness of oxic layer around the rhizosphere, then attenuate the effects of ROL on sediment redox potential, so that the nitrification may be compensated by an increased respiratory demand [51,54]. Thus, compared with P-SMFC-o, the facilitated OM degradation under electrogenesis increased the influence of ROL on nitrification, leading to a higher level of NO_3_^-^ in the pore water of P-SMFC-c.

The coupled system enhanced denitrification in sediments compared to other two treatments. Under electrogenesis, the EAB might compete with the facultative denitrifier *Rhodoplanes* for putative short-chain fatty acids, leading to a lower abundance of *Rhodoplanes* at the anodes compared to P-SMFC-o, however, the electricity significantly enriched *Bacillus* and *Pseudomonas* [55] as the heterotrophic denitrifiers at the anodes of P-SMFC-c. Compared to SMFC-c, the ROL of *V*. *spiralis* significantly elevated the abundance of the nitrifier *Nitrospirae* and aerobic denitrifier *Pseudomonas* on the anodes, implying a stronger nitrification-denitrification of sediments in P-SMFC-c. The plant root exudates and litter decomposition might serve as carbon sources for denitrification, reflected by the anode microbial community structure. Typically, the analyses of bacterial communities on electrogenic biofilms in MFCs fed with acetate and propionate have shown the dominance of *Geobacter*-related species, which can directly deliver electrons to anodes [36,56]. The short-chain fatty acids, such as lactate, acetate, and propionate, excreted by the roots of *V*. *spiralis* could result in a higher abundance of *Geobacter*. A similar result showing the predominance of *Geobacter* on the anode surface in a P-SMFC constructed by rice plants was previously reported [57]. *Clostridium*, including a variety of carbohydrate-fermenting organisms, has been reported to have syntrophic interactions with *Geobacter* for electricity generation from cellulose [58]. *Bacteroidetes*, known to be very efficient in degrading biopolymers such as cellulose and chitin [59], were enriched on the anodes of P-SMFC-c. These results imply that cellulose is prone to accumulate when aquatic plants are present, the followed fermentation of cellulose may lead to an increase in acetate as the end-product for power generation. This interactive process between the plant rhizosphere and electrogenesis also promoted the denitrification.

SMFC and *V*. *spiralis* played a complementary role in the upper and bottom layers and the best removal effect was achieved in the middle layer by P-SMFC-c. P-SMFC-c constantly showed maximum internal TN removal and overcame the defects of P-SMFC-o and SMFC-c constrained by plants and electrogenesis. Internal TN values were reduced by 8.1%, 16.2%, 24.7%, and 25.3% compared to SMFC-o on the 55th, 82th, 136th, and 190th days, respectively. These results suggest that simultaneous introduction of closed-circuit SMFC and submerged aquatic plants to inhibit the release of internal N is a feasible process.

Limited by various conditions, there is a still lack of understanding of the mechanisms between the plant rhizosphere and electrogenesis from all aspects of water, sediments, aquatic plants, and gases. Future studies should focus on the complex interactions between SMFC and aquatic plants from the perspective of rhizosphere and microorganisms. In practice, the effect of the coupled system on internal TN removal is restricted by electrogenesis, therefore, the methods to prolong the cycle and the capacity of power production should be further studied. Similar to the single phytoremediation, the effects of the coupled system were stronger in the upper and middle layers. If aquatic plants with longer root lengths are chosen, the closed-circuit SMFC in the bottom will have a stronger influence on internal TN removal.

## Conclusions

The decrease of ON in sediments dominated the internal N removal in the SMFC and P-SMFC systems. Both electrogenesis and aquatic plants could facilitate the mineralization of organic nitrogen in sediments.

The closed-circuit SMFC increased the N level in the overlying water, although a facilitated internal N removal was observed. The elevated pH value in the overlying water caused by electrogenesis boosted the volatilization of NH_4_^+^ from sediments to overlying water. This process further resulted in the accumulation of NH_4_^+^ followed by the transformation to NO_3_^-^ in the overlying water. The increased thickness of the oxidized layer of sediments due to the introduction of anode also facilitated nitrification in pore water and inhibited denitrification at the sediment-water interface.

When the aquatic plants were introduced into the closed-circuit SMFC, mineralization of internal ON in the coupled system was the strongest. The assimilation of internal NH_4_^+^ by aquatic plants under electrogenesis was advanced and nitrification in pore water and denitrification in sediments were also promoted, resulting in the the highest decrease in internal N with low N levels in the overlying water.

The lower abundance of nitrifiers, and significantly enriched denitrifying bacteria with the ability to generate power on the anodes under closed circuit might explain the improved mineralization and denitrification in sediments.

## Supporting information

S1 Fig Changes in (a) DO (b) pH, and (c) T in the overlying water over time. (TIF)

S1 Table pH of the sediments after the experiment. The data was presented as mean value ± standard deviation.
(PDF)

S2 Table Biomass and nitrogen contents of aquatic plants in P-SMFC-o and P-SMFC-c before and after the experiment. The data was presented as mean value ± standard deviation. b and a represent before and after the experiment, respectively.
(PDF)

S3 Table The class level distribution of the most dominant phylum of *proteobacteria* from communities of anode biofilms in SMFCs and P-SMFCs. (PDF)
