# Supplementary figures and images for: Internal nitrogen removal from sediments by the hybrid system of microbial fuel cells and submerged aquatic plants
Source: PLoS One. 2017 Feb 27;12(2):e0172757. doi: 10.1371/journal.pone.0172757 (PMC5328281; doi:10.1371/journal.pone.0172757)

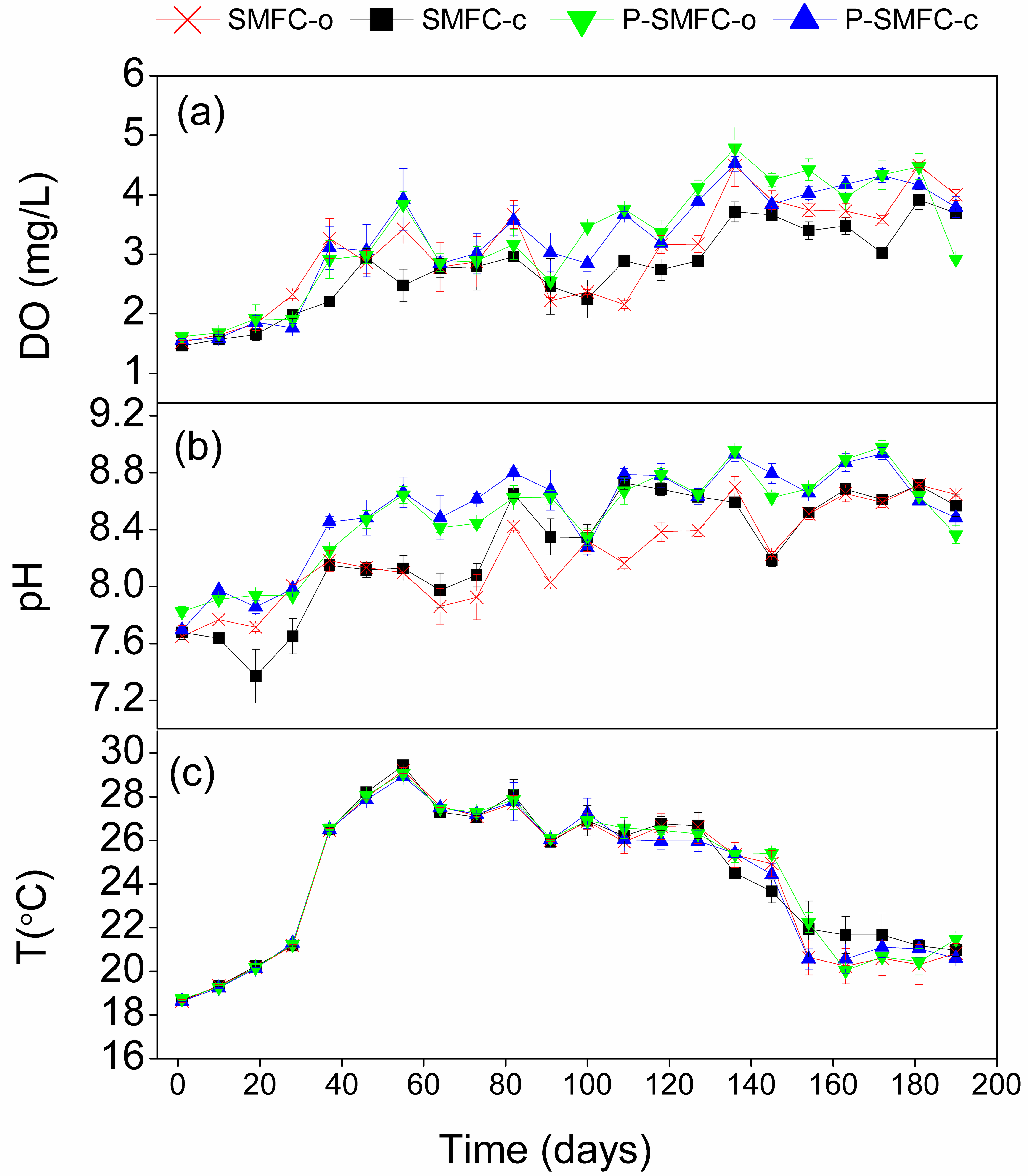

Supplement: S1 Fig — (TIF) [file pone.0172757.s001.tif]
