# Supplementary material for: Internal nitrogen removal from sediments by the hybrid system of microbial fuel cells and submerged aquatic plants
Source: PLoS One. 2017 Feb 27;12(2):e0172757. doi: 10.1371/journal.pone.0172757 (PMC5328281; doi:10.1371/journal.pone.0172757)
Supplement: S1 Table — The data was presented as mean value ± standard deviation. (PDF) [file pone.0172757.s002.pdf]

S1 Table

	SMFC-o	SMFC-c	P-SMFC-o	P-SMFC-c
pH	7.21 ±0.36	6.89 ±0.35	6.63 ±0.40	6.26 ±0.36
