# Supplementary material for: Internal nitrogen removal from sediments by the hybrid system of microbial fuel cells and submerged aquatic plants
Source: PLoS One. 2017 Feb 27;12(2):e0172757. doi: 10.1371/journal.pone.0172757 (PMC5328281; doi:10.1371/journal.pone.0172757)
Supplement: S2 Table — The data was presented as mean value ± standard deviation. b and a represent before and after the experiment, respectively. (PDF) [file pone.0172757.s003.pdf]

S2 Table

Experimental group	Fresh weight(b) (g)	Fresh weight(a) (g)	Dry weight(a) (g)	Nitrogen content(a) (mg)
P-SMFC-o	104.9 ±9.3	391.2 ±26.4	25.9 ±2.2	243.7 ±12.5
P-SMFC-c	107.5 ±17.4	412.6 ±30.7	23.4 ±1.5	291.0 ±22.8
