# Supplementary material for: Internal nitrogen removal from sediments by the hybrid system of microbial fuel cells and submerged aquatic plants
Source: PLoS One. 2017 Feb 27;12(2):e0172757. doi: 10.1371/journal.pone.0172757 (PMC5328281; doi:10.1371/journal.pone.0172757)
Supplement: S3 Table — (PDF) [file pone.0172757.s004.pdf]

S3 Table

Taxonomy	*a-proteobacteria*	*β-proteobacteria*	*γ-proteobacteria*	*δ-proteobacteria*
SMFC-o	0.013	0.118	0.0872	0.1305
SMFC-c	0.008	0.0568	0.0679	0.1517
P-SMFC-o	0.0219	0.1399	0.1002	0.1609
P-SMFC-c	0.0051	0.0491	0.0483	0.2479
